# Case Report: The acute appendicitis and incarcerated umbilical hernia: a rare association

**DOI:** 10.3389/fped.2024.1334562

**Published:** 2024-02-06

**Authors:** Letizia Corbi, Simone Frediani, Ivan Pietro Aloi, Arianna Bertocchini, Antonella Accinni, Valerio Pardi, Alessandro Inserra

**Affiliations:** General and Thoracic Pediatric Surgery Unit, Bambino Gesù Children’s Hospital, IRCCS, Rome, Italy

**Keywords:** appendicitis, peritonitis, children, umbilical hernia, acute abdominal

## Abstract

**Introduction:**

One of the most prevalent congenital wall abnormalities in children, umbilical hernias are often linked to premature or small-for-gestational-age babies. In cases of intestinal malrotation or if the cecum is very movable, generalized peritonitis may facilitate the imprisonment of these hernias.

**Case report:**

We described a case of a 4-month-old baby who had a prior reducible umbilical hernia with a history of fever, vomiting, poor appetite, and constipation for around 48 h. The patient experienced significant intestinal bloating, vomiting, irreducibility of the umbilical hernia, skin pigmentation, and erythema at the umbilical site within 2 days after hospitalization. When there was no free abdominal air, a direct abdominal x-ray revealed evidence of hydro-gas stasis and various hydro-aerial levels that were pertinent to the ileum. In order to reduce the hernia, the patient had an emergency surgical treatment where the hernia sac was isolated and released from the ileal loop, which was securely attached to a fibrin plate. When the herniary sac was opened, a gangrenous and perforated appendix was found inside. On the seventh postoperative day, the patient was released from the hospital after an uncomplicated postoperative stay.

**Conclusion:**

Our patient's clinical presentation is similar to that of only one other case report involving a 25-day-old male patient. Our case presented with a variant of the clinical symptoms of the previously described umbilical hernia, which became unfixable and strangulated as a result of appendix inflammation. The appendix was discovered inside the hernia sac during surgery.

## Introduction

Umbilical hernia is one of the most common congenital wall defects in children and is frequently associated with premature or small-for-gestational-age infants (1). A fascia defect at the umbilical level allows preperitoneal fat tissue, omentum, or the small or large intestine to protrude through the defect (2). Surgical treatment is rarely indicated unless there is incarceration or strangulation, which is more common for defects greater than 1.5 cm according to Lassaletta's classification (3). Generalized peritonitis can promote incarceration of these hernias, especially under conditions of intestinal malrotation or if the cecum is particularly mobile (4).

## Case report

A 4-month-old infant with a history of fever, vomiting, poor appetite and constipation for approximately 48 h, along with a previously reducible umbilical hernia, born at 34 weeks via cesarean section due to premature rupture of membranes in a twin pregnancy, was transferred to the emergency department of our institution.

**Figure 1:**
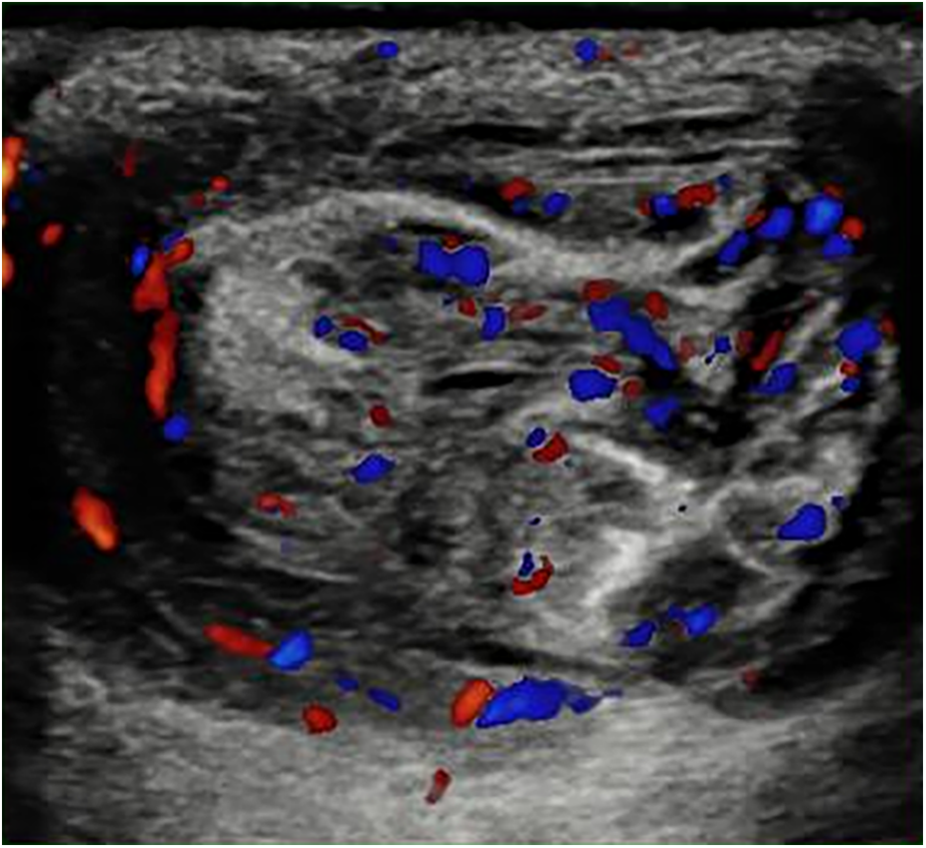
Abdominal ultrasound executed in the emergency department showing an herniated loop at umbilical site with no vascular abnormality signals on Doppler at patient presentation.

On admission, hematochemical examination showed a white blood cell count of 19.7 × 10^3^/ul, 55.9% neutrophils, a C-reactive protein of 13.77 mg/dl, and a procalcitonin level of 5.34 ng/ml. At physical examination, the patient presented abdominal distention, and an erythematic area around the umbilicus with a palpable reducible soft tissue mass within the umbilical defect. A complete abdominal ultrasound performed in the emergency department revealed diffuse and marked distention of the intestinal loops, the absence of intestinal effusion or invagination, and the presence of a reducible herniated loop at the umbilical site with no vascular abnormalities on doppler (Figure 1).

**Figure 2:**
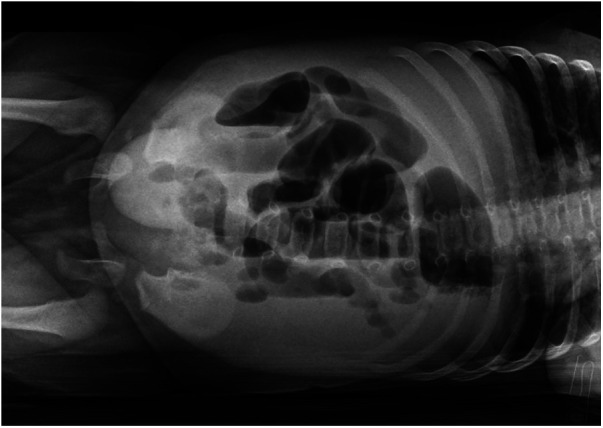
Direct abdominal x-ray performed after clinical worsening suggesting intestinal occlusion with multiple aereal levels and hygro-gas stasis.

**Figure 3:**
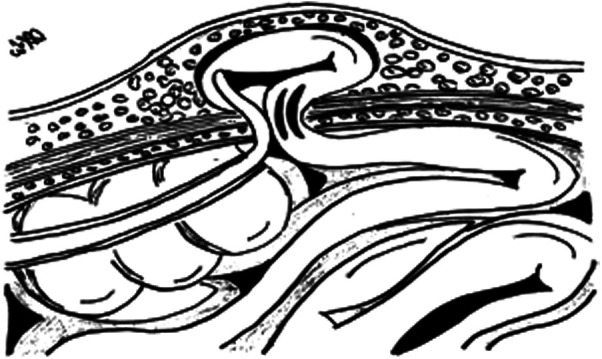
An illustrated image showing intraoperative findings with the gangrenous appendix located in the herniary sac. The skin incision is a semilunar incision (U-shaped) below the umbilical scar but we needed to expand the latter with a median longitudinal cut towards the pelvic bone in order to control the intestinal loops for ischemic areas.

Within 2 days of admission, the patient became distressed and developed severe intestinal bloating, vomiting, irreducibility of the umbilical hernia, and skin discoloration and erythema at the umbilical site. A direct abdominal x-ray showed signs of hydro-gas stasis and multiple hydro-aerial levels that were relevant to the ileum when there was no free abdominal air (Figure 2). The patient underwent an urgent surgical procedure for hernia reduction, involving the isolation and release of the hernia sac from the ileal loop, which was firmly adhered to a fibrin plate. Upon opening the herniary sac, the presence of a gangrenous and perforated appendix within it was discovered. An appendectomy was therefore performed on double tobacco-pouch technique (5, 6), and the abdominal wall defect was sutured (Figure 3). The cecal appendix and the herniary sac were sent for histological examination, which revealed a picture of acute appendicitis with acute necrotizing-exudative serositis, approximately 5 cm in size, with perforation of the appendiceal wall. The postoperative course was uneventful, and the patient was discharged on the seventh postoperative day.

## Discussion

Acute appendicitis that happens along with an umbilical hernia may be caused by an ischemic event. This happens when the appendix lumen is compressed at the herniary sac's entrance because of intestinal loops or repeated trauma that closes off the nutrient vessels. This can cause lymphatic stasis, promote appendiceal distension, and eventually lead to the development of ischemia and wall necrosis (7). Currently, given the paucity of reported cases, there are no scientific data to support the direct correlation between the location of the appendix within the hernia sac and the development of appendiceal inflammation. Although the anatomical location of the cecal appendix is generally described as at the confluence of the taeniae of the cecum, in many cases, its location can be variable and found in subhepatic or retrocecal positions.

In association with significant umbilical defects according to Lassaletta's classification and a mobile cecum, the presence of intestinal inflammatory processes can lead to an increase in peristaltic waves, resulting in the migration of the cecal appendix into the hernia sac alongside the small intestine. This can lead to the development of adhesions, favour local inflammatory conditions, and bacterial proliferation, ultimately turning a reducible umbilical hernia with preserved blood supply into an incarcerated umbilical hernia.

In contrast to the adult population where the diagnosis can often be supported by imaging such as computed tomography, in the pediatric population, efforts are made to avoid the use of ionizing radiation, and the most widely used instrumental examination is abdominal ultrasound or abdominal radiography. This can lead to a delay in diagnosis and a more challenging preoperative planning, making it possible to confirm clinical suspicion only intraoperatively (8).

There is only one other case report of a 25-day-old male patient with a similar clinical presentation to ours. The patient had a variation in the clinical features of the previously mentioned umbilical hernia that became impossible to fix and strangulated due to inflammation of the appendix. Intraoperatively, the appendix was found within the hernia sac (9).

Normally, due to the high tendency for spontaneous closure, conservative treatment is recommended for umbilical hernias in the pediatric population. Only in 0.07%–0.3% of cases do strangulation and incarceration of hernias seem to occur in infants under 6 months of age with a moderate defect according to the Lassaletta's classification (0.5–1.5 cm) (10–13). The likely pathogenesis responsible for the association and concomitance of these two pathological events seems to be related to appendiceal ischemia caused by compression at the hernia sac's entrance due to the concomitant presence of intestinal loops or recurrent trauma causing adhesions, inflammation, and bacterial proliferation (9).

## Conclusion

In the literature, eleven cases of incarcerated umbilical hernias containing an inflamed cecal appendix have been described, with only one case reported in the pediatric population. Certainly, an incarcerated appendix within a hernia sac presents challenging diagnostic and therapeutic issues. Special appendiceal conditions have been previously described within inguinal (Amyand's) or femoral (De Garengeot's) hernias (14–16), with an incidence of 1.6% in the adult population. However, the reason why acute appendicitis can develop within an umbilical hernia is unknown, and it is an extremely rare finding for which the incidence rate in children is not known. Some anatomical variations that alter or disrupt the relationship between the cecum and the peritoneum may render the appendix more mobile (4).

Variable degrees of intestinal rotation and heterogeneous cecal insertions allow the appendix to be found in anatomically unusual sites and, consequently, within hernia sacs (17, 18).

Although acute appendicitis in infants is rare due to various environmental and constitutional factors, given this rare complication, the previously described scenario should be considered in the differential diagnosis, especially in patients who present variations in the size and characteristics of a previously reducible umbilical hernia (19).

## Data Availability

The original contributions presented in the study are included in the article/Supplementary Material, further inquiries can be directed to the corresponding author.
